# Costos directos de la infección adquirida en la comunidad por neonatos a término con bajo riesgo al nacer, Cundinamarca, Colombia

**DOI:** 10.7705/biomedica.5196

**Published:** 2020-09-22

**Authors:** Sergio Iván Agudelo, Carlos Federico Molina, Óscar Andrés Gamboa, Juan David Suárez

**Affiliations:** 1 Escuela de Graduados, Universidad CES, Medellín, Colombia; 2 Departamento de Pediatría, Clínica Universidad de La Sabana, Universidad de La Sabana, Chía, Colombia; 3 Departamento de Investigación, Universidad de La Sabana, Chía, Colombia; 4 Facultad de Medicina, Universidad de La Sabana, Chía, Colombia

**Keywords:** sepsis neonatal, costos y análisis de costo, recién nacido, unidades de cuidado intensivo neonatal, mortalidad infantil, Neonatal sepsis, costs and cost analysis, infant, newborn, intensive care units, neonatal, infant mortality

## Abstract

**Introducción:**

El 50% de los episodios de sepsis neonatal se originan en la comunidad, con un gran porcentaje de mortalidad y complicaciones.

**Objetivo:**

Estimar los costos directos de la hospitalización por infección neonatal adquirida en la comunidad en neonatos a término con bajo riesgo al nacer.

**Materiales y métodos:**

Se utilizó la perspectiva del tercer pagador y la técnica de microcosteo; el horizonte de tiempo fue la duración de la hospitalización. La determinación de las situaciones generadoras de costos se obtuvo por medio de un consenso de expertos y se cuantificaron con base en la factura detallada de la atención de 337 neonatos hospitalizados. Los costos de los medicamentos se calcularon con base en el Sistema de Información de Precios de Medicamentos (SISMED) y, el de los procedimientos, según los manuales tarifarios ISS 2001 con porcentaje de ajuste y el seguro obligatorio de accidentes de tráfico (SOAT). Para incorporar la variabilidad de la información en la estimación, se obtuvo una distribución de los costos usando el método de *bootstrapping*.

**Resultados:**

Se incluyeron las facturas por la atención de 337 recién nacidos. El promedio de costos directos de la atención por paciente fue de COL$ 2’773.965 (desviación estándar, DE=$ 198.813,5; IC_95%_: $ 2’384.298 - $ 3’163.632). Las principales categorías generadoras de costos fueron la internación en la unidad de cuidados intensivos y las tecnologías en salud. Los costos siguieron una una distribución logarítmica normal (log-normal).

**Conclusiones:**

Las categorías con mayor impacto en los costos fueron la internación en la unidad neonatal y las tecnologías en salud. Los costos se ajustaron a una distribución logarítmica normal.

## Abstract (en)

**Introduction:**

Half of the episodes of neonatal sepsis are acquired in the community with a high percentage of mortality and complications.

**Objective:**

To estimate the direct costs of hospitalizations due to systemic neonatal infection acquired in the community in low-risk newborns.

**Materials and methods:**

For the estimation of costs, we used the perspective of the health systems and the microcosting technique and we established the duration of hospitalization as the time horizon. We identified cost-generating events through expert consensus and the quantification was based on the detailed bill of 337 hospitalized newborns diagnosed with neonatal infection. The costs of the medications were calculated based on the drug price information system (SISMED) and the ISS 2001 rate manuals adjusting percentage, and the mandatory insurance rates for traffic accidents (SOAT). We used the bootstrapping method for cost distribution to incorporate data variability in the estimate.

**Results:**

We included the medical care invoices for 337 newborns. The average direct cost of care per patient was USD$ 2,773,965 (Standard Deviation, SD=USD$ 198,813.5; 95% CI: $ 2,384,298 - $ 3,163,632). The main cost-generating categories were hospitalization in intensive care units and health technologies. The costs followed a log-normal distribution.

**Conclusions:**

The categories generating the greatest impact on the care costs of newborns with infection were hospitalization in neonatal units and health technologies. The costs followed a log-normal distribution.

Todos los autores participaron en la concepción y el diseño del estudio, el análisis y la interpretación de los datos, y la redacción del artículo.

La morbilidad en los recién nacidos a término no es una situación infrecuente, como tampoco lo es la hospitalización en el primer mes de vida (1-3). Además, la infección neonatal adquirida en la comunidad por recién nacidos con bajo riesgo al nacer, es la principal causa de morbilidad y mortalidad en países con ingresos económicos medios y bajos, y produce el mayor número de ingresos a las unidades neonatales (4-6). En estos países, un importante porcentaje de la sepsis neonatal adquirida en la comunidad es causado por bacterias resistentes, lo que se ha convertido en un problema de salud pública mundial por las complicaciones que implica y por el incremento en el costo de la atención en salud (7). En Colombia, ha habido un aumento de la morbilidad y la mortalidad neonatal temprana de recién nacidos a término con bajo riesgo, principalmente por causas prevenibles, incluida la infección neonatal (8).

La infección neonatal sistémica adquirida en la comunidad agrupa condiciones clínicas que se presentan en los primeros 28 días de vida, como infección respiratoria, sepsis, bacteriemia, infección urinaria, meningitis, diarrea y onfalitis (7,9). En las regiones con ingresos económicos bajos y medios, cerca del 50% de todos los episodios de sepsis neonatal son adquiridos en la comunidad y, de ellos, el 16% se presenta con bacteriemia neonatal y con mayor tasa de mortalidad (4). En estos países se reporta una tasa anual de 0,66 a 1,03 por 1.000 nacidos vivos (10).

En países con ingresos bajos y medios, es difícil el acceso a unidades neonatales pues en ellas la atención médica es de alto costo, lo que complica el curso y el resultado de la infección neonatal adquirida en la comunidad; esta situación contrasta con la disponibilidad de intervenciones preventivas enfocadas a disminuir las infecciones en este grupo de neonatos (1,11). Asimismo, se estima que el costo diario de atención en las unidades neonatales puede ser dos a tres veces mayor que el del que se brinda en salas generales (12). Por otro lado, las infecciones en el neonato tienen efectos a corto y largo plazo en su salud, lo que impone una carga económica importante para la familia, la sociedad y los sistemas de salud (13,14).

Los recursos de los sistemas de salud son limitados y compiten con otros objetivos sociales también cruciales para la población (15). Las evaluaciones económicas en salud son importantes para asignar los recursos, pues aportan a los responsables de las decisiones información objetiva basada en los datos sobre la efectividad y los costos de las nuevas tecnologías, que reduce la incertidumbre al decidir (14,15).

Uno de los elementos de las evaluaciones económicas son los costos de la atención, pero los estudios económicos sobre la atención neonatal son escasos. En Colombia, hay poca información sobre los costos de la atención en las unidades neonatales de enfermedades prevalentes y prevenibles en el neonato a término con bajo riesgo al nacer, y no se encontraron estudios de costos de la atención de las infecciones neonatales sistémicas en este grupo.

Por ello, en este estudio se propuso estimar los costos directos de la atención del recién nacido a término con bajo riesgo y con diagnóstico de infección neonatal sistémica adquirida en la comunidad, en una unidad de cuidado neonatal de una clínica universitaria que atiende a población de Sabana Centro, provincia de Cundinamarca. Esta información podría ser útil para determinar los costos totales en que incurre el sistema de salud y ayudaría a estimar el ahorro que se haría si se reduce su prevalencia mediante la implementación de intervenciones preventivas. Además, serviría para hacer evaluaciones económicas en salud más complejas.

## Materiales y métodos

### Diseño

Se estimaron los costos por enfermedad desde la perspectiva del tercer pagador, incluidos los directos, es decir, los correspondientes al personal de salud, la estancia hospitalaria y el uso de tecnologías en salud (medicamentos, procedimientos, dispositivos e insumos), así como los costos de la atención según los niveles de gravedad, pero no se incluyeron aquellos derivados de las secuelas crónicas de la infección neonatal.

### Población

Se obtuvo información de los recién nacidos hospitalizados entre noviembre de 2006 y junio de 2017 en la unidad neonatal de una clínica universitaria que atiende a la población del régimen contributivo de la provincia de Sabana Centro (11 municipios de Cundinamarca).

Se incluyeron recién nacidos a término con peso adecuado para la edad gestacional (entre 37 y 41 semanas cumplidas, según el test de Ballard y peso entre el percentil 10 y el 90 para la edad gestacional), dados a luz en parto vaginal y con adaptación neonatal espontánea; que cumplían los criterios de bajo riesgo al nacer y que fueron hospitalizados en la unidad neonatal después del egreso de salas generales y hasta los 30 días de vida; con ingreso a la unidad de sepsis neonatal por diagnóstico de infección neonatal sistémica (que incluyó infección neonatal, neumonía, meningitis, sepsis, infección urinaria y bacteriemia); con un cuadro clínico que hubiera requerido antibióticos y, finalmente, con síntomas o signos de infección neonatal sistémica que son disminución en la capacidad de alimentación, ausencia de movimientos espontáneos, temperatura mayor de 38 °C, alteración del estado de conciencia, problemas de alimentación, cambios en la actividad, agitación, tiraje intercostal bajo, frecuencia respiratoria mayor de 60 por minuto, quejido, cianosis, convulsión, fontanela abombada y llenado capilar lento, según la Organización Mundial de la Salud (OMS) (5).

Se excluyeron los recién nacidos con factores de riesgo perinatales al nacer (hijos de madres con complicaciones médicas, obstétricas o neonatales), aquellos con malformaciones congénitas y los que hubieran requerido el ingreso a la unidad neonatal inmediatamente después del nacimiento o antes del egreso de salas generales.

### Estimación de costos

Los costos se estimaron a partir de la base de datos de recién nacidos atendidos en la unidad de cuidado neonatal de una clínica universitaria entre noviembre de 2006 y junio de 2017, y que cumplían con los criterios de inclusión en el estudio. La estimación se hizo por microcosteo en tres fases: determinación, cuantificación y valoración.

*Determinación de los eventos generadores de costos*. Estos se categorizaron en, 1) tecnologías en salud: exámenes de laboratorio e imágenes diagnósticas, procedimientos, medicamentos y dispositivos; 2) consultas: apoyo terapéutico en nutrición, terapia física y terapia respiratoria, entre otras, e interconsulta con especialidades médicas, y 3) tipo de internación en la unidad neonatal: cuidado básico, intermedio o intensivo.

Dado que en nuestro medio no se dispone de guías basadas en la “evidencia” para la atención del neonato a término con riesgo bajo e infección neonatal sistémica, para determinar los costos de la atención en cada categoría se recurrió al consenso de opinión entre expertos aplicando el método Delphi, como se describe a continuación:

• Expertos: participaron diez neonatólogos con cinco o más años de experiencia profesional en unidades de cuidado neonatal de mediana y alta complejidad. Se tuvo la intención de incluir jefes de unidades neonatales y profesores universitarios.

• Rondas del consenso: en las rondas programadas se mantuvo en todo momento el anonimato de los participantes, y se utilizó el correo electrónico para el envío y la recepción de las encuestas.

En la primera, se envió un cuestionario con preguntas abiertas, planteándole a los expertos diferentes situaciones clínicas de condiciones asociadas con la infección neonatal sistémica y de diversa gravedad. Se les solicitó que, con base en su experiencia, plantearan en cada caso las diferentes tecnologías en salud y consultas que solicitarían, así como el tipo de atención que podría requerir el neonato. Las respuestas de esta primera ronda fueron analizadas y resumidas por los investigadores para establecer las primeras categorías. Con estos resultados iniciales, se estructuró una encuesta con preguntas de tipo de la escala de Likert (totalmente de acuerdo, de acuerdo, parcialmente de acuerdo y en desacuerdo).

Esta encuesta se envió en la segunda ronda del proceso conjuntamente con la opinión de los otros encuestados sobre cada pregunta como retroalimentación sobre. Se estableció *a priori* que el consenso se alcanzaría con un 80% o más de respuestas “de acuerdo y totalmente de acuerdo” para incluir un ítem y “en desacuerdo” para excluirlo. Se planificaron tres rondas para establecer el consenso.

*Cuantificación.* Una vez determinadas las categorías de eventos generadores de costos mediante el consenso, se hizo la cuantificación con base en la factura detallada de la atención de los pacientes ingresados a la unidad de cuidado neonatal de la clínica universitaria.

*Valoración de los recursos.* Se usó el costo unitario del medicamento estimado a partir de la base del SISMED y la Circular 04 del 2018. Para valorar el consumo de medicamentos, se multiplicó su precio unitario por la cantidad promedio utilizada. El costo de los recursos utilizados en los procedimientos se calculó usando el manual tarifario ISS 2001, más un porcentaje de ajuste del 30% para el mínimo y de 42% para el de base y otro según el SOAT para el máximo.

Para estimar los costos de los medicamentos según la base del SISMED (2018), se siguieron los siguientes pasos,

1. Se extrajo la información sobre los medicamentos que no tenían topes de precio en la Circular 04 de 2018.

2. Dado que un mismo principio activo puede tener varias presentaciones y ser producido por diferentes compañías farmacéuticas, se estimó el costo ponderado por dosis del medicamento usando como ponderador las unidades reportadas.

3. Se usó la información del canal institucional.

4. El precio de base, el mínimo y el máximo correspondieron a los reportados en el SISMED; para los medicamentos con precio tope, este se usó como el máximo.

El costo ponderado se calculó con la siguiente fórmula:

,

donde *C_l_* es el costo por dosis ponderado para el medicamento *l*; *m* es el número de presentaciones del medicamento (definidas según los miligramos por tableta, vial, etc., y la marca, pues una misma marca podría tener más de una presentación); *ci* es el costo por dosis de la iésima presentación del medicamento *l* con *i = 1, 2,*. . .*m*; *mi* es el número de unidades reportadas para la iésima presentación del medicamento *l* con *i = 1, 2,*. . .*m*, y *n* es el total de unidades reportadas para el medicamento *l*. En los medicamentos con precio tope por miligramo, se usó la información reportada en la circular 04 de 2018.

### Análisis estadístico

Los costos totales de la infección neonatal sistémica para cada paciente se estimaron como se muestra a continuación:

,

donde es el número de procedimientos (diagnósticos o terapéuticos) o medicamentos para una condición determinada *E*; es el costo del iésimo procedimiento o medicamento, y es la cantidad del iésimo procedimiento o medicamento.

Los costos totales para cada condición se presentan según la información de las tarifas ISS con ajuste del 30% para el precio mínimo, de 42% para el de base y, para el máximo, el SOAT, de los medicamentos contemplados en el canal institucional de la base SISMED.

Se hicieron análisis descriptivos de los costos usando medidas de tendencia central (promedio, mediana) y de dispersión (desviación estándar y rangos). Para incorporar la variabilidad de la información en la estimación, se obtuvo una distribución de los costos mediante el método de *bootstrapping*, que consiste en un proceso de remuestreo con reemplazamiento, con el cual se generaron 10.000 registros a partir de la información disponible a nivel individual. El estimador de la media poblacional se definió como:

,

y con un error de muestreo:donde Tn (.) = estimador *bootstrap* de la media poblacional; X = población; B = tamaño de la muestra, y Tn (Xb) = remuestreo *bootstrap* (valor obtenido para el costo en cada muestreo).

Los costos se estimaron en pesos colombianos (COP) del 2019 convertidos a dólares estadounidenses (USD) con base en el promedio de la tasa de intercambio establecida por el Banco de la República para el 2019. Los análisis se llevaron a cabo utilizando Microsoft Excel 2016™ y Stata 14™.

## Resultados

### Conformación del panel de expertos y determinación de eventos generadores de costos

Se invitó a participar en el panel de expertos a diez neonatólogos que cumplían con la definición de experto y, finalmente, se contó con la colaboración de nueve que completaron todas las rondas hasta llegar al consenso. Todos tenían diez o más años de experiencia en atención de recién nacidos en unidades neonatales y estaban vinculados a hospitales de mediana y alta complejidad: ocho, a hospitales universitarios, cinco, a hospitales privados, y cuatro, a hospitales públicos de la red del Distrito de Bogotá o la gobernación de Cundinamarca. Cuatro expertos ejercían como jefes o coordinadores de unidades neonatales y ocho eran profesores universitarios. El consenso sobre las categorías de los eventos generadores de costos, se alcanzó totalmente en todas las categorías en la tercera ronda y se presentan en el cuadro 1.

**Cuadro 1:** Eventos generadores de costos definidos por el consenso de expertos

**Tecnologías en salud**			
Antibióticos	Ampicilina, penicilina cristalina, ampicilina, sulbactam, piperacilina, tazobactam, vancomicina, cefalotina, cefotaxima, cefepime, amikacina, gentamicina, claritromicina	Otros medicamentos	Acetaminofén, líquidos intravenosos (lactato de Ringer, solución salina, soluciones de dextrosa), oxígeno, inotrópicos (dopamina, dobutamina, norepinefrina, adrenalina, milrinona), dipirona, fenobarbital, fenitoína, cloruro de potasio, cloruro de sodio, dexametasona
Exámenes de laboratorios	Química sanguínea, microbiología: cultivos Gram, látex, gases sanguíneos, uroanálisis, antígenos virales (VSR, influenza, adenovirus, parainfluenza)	Imágenes diagnósticas	Radiografía convencional de tórax y abdomen, ecografía, tomografía axial, resonancia magnética cerebral
Procedimientos	Médicos (punción lumbar, intubación orotraqueal, punción suprapúbica) Terapia (nebulización)	Insumos y dispositivos	Nutrición parenteral Asistencia respiratoria mecánica no invasiva (CPAP- cánula nasal de alto flujo) Asistencia respiratoria mecánica invasiva Catéter central Catéter umbilical Catéter epicutáneo Catéter heparinizado
**Internación en la unidad neonatal**			
Cuidado básico Cuidado intermedio		Cuidado intensivo Aislamiento respiratorio	
**Consultas**			
Medicina especializada Neurología pediátrica Neurocirugía Cirugía pediátrica		Terapia respiratoria Terapia física Fonoaudiología Fisiatría	

CPAP: *Continuous positive airway pressure therapy*

### Costos directos de la atención

**Figura 1:**
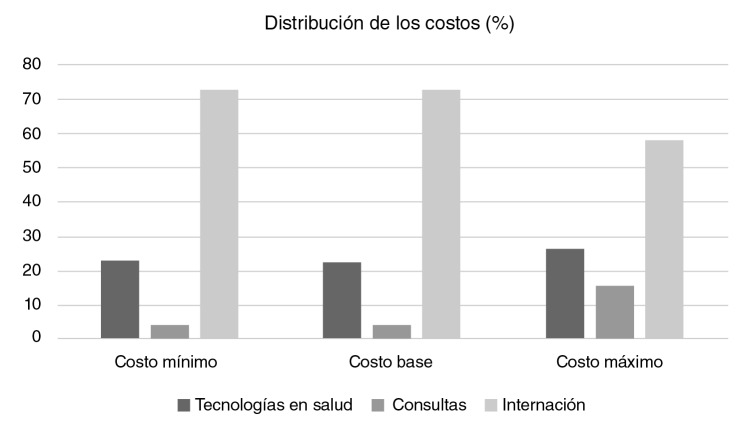
Distribución de los costos directos asociados con la infección neonatal adquirida en la comunidad en recién nacidos con bajo riesgo al nacer

Se incluyeron 337 recién nacidos en el estudio y el costo directo de base promedio de la infección neonatal sistémica adquirida en la comunidad en recién nacidos con bajo riesgo, fue de COP$ 2’773.965 (USD$ 860) y el costo total en el período de estudio fue de COP$ 530’518.016. Los costos directos estimados se presentan en el cuadro 2. La categoría asociada con los mayores costos fue la internación en la unidad neonatal (73% de los costos directos), con un promedio de costos directos de base de COP$ 2’029.102 (USD$ 629,42), principalmente por la necesidad de internación en la unidad de cuidado intensivo (COP$ 1’155.361; USD$ 358,39). En el cuadro 3 y la figura 1, se presenta la distribución de los costos por categorías de eventos generadores de costo, entre los cuales los principales fueron los exámenes de laboratorio y las imágenes diagnósticas (promedio del costo directo de base: COP$ 330.176; USD$ 114,81), seguidos por los dispositivos médicos. La categoría que menos generó costos fue la de los medicamentos.

**Cuadro 2:** Costos directos de atención médica por infección neonatal sistémica adquirida en la comunidad en recién nacidos a término y de bajo riesgo

	**Costo mínimo**	**Costo de base**	**Costo máximo**
Promedio	$ 2’580.278	$ 2’773.965	$ 3’488.736
DE	$ 3’394.772	$ 3’675.405	$ 3’986.253
Mediana	$ 1’413.131	$ 1’455.545	$ 2’151.665
RIC	$ 2’274.573	$ 2’492.374	$ 2’771.826
Percentil 25	$ 744.270	$ 775.867	$ 1’295.044
Percentil 75	$ 3’018.843	$ 3.268.242	$ 4’066.871
Mínimo	$ 128.108	$ 141.386	$ 260.010
Máximo	$ 26’510.728	$ 28’664.677	$ 30’510.190

DE: desviación estándar; RIC: rango intercuartílico

**Cuadro 3:** Costo promedio por categoría generadora de costo

	**Costo mínimo**	**Costo de base**	**Costo máximo**
Tecnologías en salud			
Antibióticos	$ 6.522	$ 7.461	$ 9.031
Otros medicamentos	$ 29.447	$ 38.908	$ 57.258
Exámenes de laboratorios e imágenes diagnósticas	$ 302.640	$ 330.176	$ 605.300
Procedimientos	$ 49.521	$ 54.092	$ 55.928
Dispositivos	$ 193.812	$ 193.812	$ 193.812
Costo total	$ 581.943	$ 624.450	$ 921.330
Consultas			
Consultas	$ 110.238	$ 120.414	$ 538.305
Internación			
Cuidad básico	$ 360.547	$ 360.547	$ 360.547
Cuidado intermedio	$ 469.825	$ 513.193	$ 513.193
Cuidado intensivo	$ 1’057.725	$ 1’155.361	$ 1’155.361
Total	$ 1’888.097	$ 2’029.102	$ 2’029.102
Total costos directos	$ 2’580.278	$ 2’773.965	$ 3’488.736

### Distribución de los costos

Utilizando el método de *boostrapping*, se obtuvo una media de COP$ 2’773.965 (DE=$ 198.813,5; IC_95%_ COP$ 2’384.298 - $ 3’163.632) y una mediana de COP$ 1’455.545 (IC_95%_ COP$ 1’213.777 - $ 1’697.313); se determinó que los costos seguían una distribución normal logarítmica (media=13,1704722; DE=1,82500197).

## Discusión

En este estudio se estimaron los costos directos de la hospitalización de neonatos con bajo riesgo al nacer por infección neonatal sistémica adquirida en la comunidad, en una cohorte de neonatos atendida en una clínica universitaria con influencia en la provincia Sabana Centro de Cundinamarca (11 municipios) y el norte de Bogotá. Estos datos son importantes para evaluaciones económicas completas de las intervenciones preventivas orientadas a esta población.

En Colombia no se han hecho estudios de este tipo que permitan la comparación con nuestros resultados. Sin embargo, la infección neonatal adquirida en la comunidad tiene una prevalencia alta y es la principal causa de hospitalización de neonatos a término y con bajo riesgo en países clasificados como de ingresos económicos medios y bajos (6). Aunque en Colombia no hay datos sobre la frecuencia de la sepsis adquirida en la comunidad en este grupo específico de recién nacidos, en el 2017 las infecciones en el periodo neonatal fueron la segunda causa de mortalidad en los menores de un mes de vida (16). En otros países se reconoce que la infección neonatal adquirida en la comunidad tiene implicaciones económicas adversas para la familia, los sistemas de salud y los países (14,17). En este contexto, los costos directos estimados en el presente estudio confirman que la enfermedad impone una gran carga económica a las familias y los sistemas de salud; además, constituyen un primer paso para evaluar la carga de la enfermedad y los posibles ahorros del sistema de salud con la implementación o ampliación de la cobertura de las intervenciones preventivas en salud neonatal.

Hasta el momento, los estudios de costos en unidades de cuidado neonatal se han enfocado en el prematuro y en el neonato en estado crítico al nacer (18). Sin embargo, la hospitalización del neonato a término no es infrecuente, siendo las infecciones en el primer mes de vida una de las causas principales (1,3). En Perú, se reportó un costo de atención por sepsis neonatal en prematuros de USD $ 928,50 en el 2004 y se estableció una relación directa del aumento del costo con la gravedad de la enfermedad (19). El costo promedio de la hospitalización del neonato a término se ha estimado en otros países en USD$ 2.900 (18), un valor por encima del obtenido en este estudio. Sin embargo, se debe tener en cuenta que se trata de neonatos de bajo riesgo al nacer, un grupo que constituye el mayor porcentaje de recién nacidos, por lo que, dada la frecuencia de la enfermedad, los costos totales para el sistema de salud y las familias pueden constituir una fuente importante de gastos.

Asimismo, aunque el costo de la unidad neonatal es inversamente proporcional al peso y la edad gestacional al nacer, el grupo de neonatos con adecuado peso al nacer genera un porcentaje importante de los costos de la unidad neonatal. Shanmugasundaram, *et al.* (20), en una unidad de cuidado intensivo neonatal de alta complejidad en la India, encontraron que los neonatos con un peso mayor de 2.500 g ocupaban el tercer lugar como grupo generador de costos, por encima de los neonatos de 2.000 a 2.499 g de peso al nacer.

En el presente estudio, los principales eventos generadores de costos fueron la internación en la unidad neonatal, en especial, por cuidados intensivos, y las tecnologías en salud (exámenes de laboratorios e imágenes diagnósticas), lo cual es similar a lo reportado por otros autores en estudios en prematuros en unidades neonatales (20,21). Sin embargo, hay información que contrasta con esta y da cuenta de que los eventos que más generan costos son los medicamentos y los insumos (22). Dado que los principales generadores de costos fueron la internación y los exámenes de laboratorio para el estudio de la enfermedad, es importante que ello se tenga en cuenta en neonatos a término con bajo riesgo, pues hay intervenciones sencillas que disminuyen la infección neonatal en este grupo (lactancia materna, contacto piel a piel, cuidado higiénico del parto, entre otros) (23); la ampliación de su cobertura en Colombia y la de otras estrategias, como el seguimiento comunitario y domiciliario del neonato, reducirían la necesidad de internación por esta enfermedad y representarían una disminución de costos para el sistema de salud (24,25), lo que constituye un desafío para la academia, las instituciones de salud y el sistema de salud.

La promoción de intervenciones sencillas y costo-efectivas, como el contacto piel a piel al nacimiento, la lactancia temprana (en la primera hora) y exclusiva, el cuidado higiénico de la atención del parto y del neonato, ha demostrado ser efectiva para disminuir la morbilidad y la mortalidad por causas infecciosas en recién nacidos (26). En este sentido, los datos del presente estudio sirven en las evaluaciones económicas completas de estas intervenciones, útiles para los responsables de las decisiones a la hora de implementar las estrategias.

Debe anotarse, además, que las complicaciones y secuelas a largo plazo de la infección neonatal derivan en una carga económica importante (17). La prevención de la infección neonatal podría evitar este impacto económico. En su estudio, Ranjeva, *et al*. (27), plantean que, si todos los casos anuales de sepsis neonatal en el África subsahariana se pudieran prevenir, podría evitarse la pérdida de entre 5,29 y 8,73 millones de años de vida ajustados por discapacidad (AVAD). La implicación económica anual de los AVAD evitados con el tratamiento o la prevención adecuada estaría en el rango de USD$ 9,3 a $ 469,15 billones.

Dado que no hay información sobre el costo total en que incurre el sistema de salud por estas enfermedades en los neonatos de bajo riesgo en el país, los resultados del presente estudio son un primer acercamiento a la estimación de la carga de la enfermedad para el sistema de salud y un incentivo para invertir en intervenciones preventivas en salud neonatal que permitan disminuir las tasas de la enfermedad. Asimismo, los datos del estudio constituyen un elemento necesario para hacer la evaluación económica completa de tales intervenciones.

El estudio tiene también limitaciones. La información para la cuantificación de los recursos se obtuvo de una cohorte retrospectiva de pacientes a partir de una base de datos, lo que podría haber introducido algún sesgo. Por otra parte, dado que en el país no hay guías sobre la sepsis en neonatos de riesgo bajo, la definición de infección se unificó a partir de los criterios de la OMS para países con recursos económicos medios y bajos, y las categorías se establecieron mediante un consenso de expertos. Asimismo, se adoptó únicamente la perspectiva del tercer pagador y el horizonte de tiempo fue la hospitalización, por lo que en estudios futuros sería importante incluir una perspectiva más amplia, como la social, y considerar resultados genéricos en salud, como los AVAD, que se emplean en los estudios de carga de la enfermedad. Se recomienda, por ello, hacer evaluaciones económicas más complejas tomando en cuenta las intervenciones preventivas en sepsis neonatal en recién nacidos de bajo riesgo, para respaldar las decisiones de inversión en salud pública.

En conclusión, el costo directo de base promedio de la infección neonatal adquirida en la comunidad en neonatos de bajo riesgo al nacer fue de COP$ 2’773.965. Las categorías que generaron la mayoría de los costos en la atención del recién nacido, fueron la internación en la unidad neonatal y las tecnologías en salud (exámenes de laboratorios e imágenes diagnósticas). Los costos se ajustaron a una distribución logarítmica normal.
